# The genetic basis and interaction of genes conferring resistance to *Puccinia hordei* in an ICARDA barley breeding line GID 5779743

**DOI:** 10.3389/fpls.2022.988322

**Published:** 2022-08-16

**Authors:** Hoan X. Dinh, Mohammad Pourkheirandish, Robert F. Park, Davinder Singh

**Affiliations:** ^1^Faculty of Science, Plant Breeding Institute, The University of Sydney, Sydney, NSW, Australia; ^2^Faculty of Veterinary and Agricultural Sciences, The University of Melbourne, Parkville, VIC, Australia

**Keywords:** barley (*Hordeum vulgare*), *Puccinia hordei*, leaf rust, complementary genes, interaction, disease resistance, mapping, QTL

## Abstract

Leaf rust of barley causes significant losses in crops of susceptible cultivars. Deploying host resistance is the most cost-effective and eco-sustainable strategy to protect the harvest. However, most known leaf rust resistance genes have been overcome by the pathogen due to the pathogen’s evolution and adaptation. The discovery of novel sources of genetic resistance is vital to keep fighting against pathogen evolution. In this study, we investigated the genetic basis of resistance in barley breeding line GID 5779743 (GID) from ICARDA, found to carry high levels of seedling resistance to prevalent Australian pathotypes of *Puccinia hordei*. Multipathotype tests, genotyping, and marker-trait associations revealed that the resistance in GID is conferred by two independent genes. The first gene, *Rph3*, was detected using a linked CAPS marker and QTL analysis. The second gene was detected by QTL analysis and mapped to the same location as that of the *Rph5* locus on the telomeric region of chromosome 3HS. The segregating ratio in F_2_ (conforming to 9 resistant: 7 susceptible genetic ratio; *p* > 0.8) and F_3_ (1 resistant: 8 segregating: 7 susceptible; *p* > 0.19) generations of the GID × Gus population, when challenged with pathotype 5477 P− (virulent on *Rph3* and *Rph5*) suggested the interaction of two genes in a complementary fashion. This study demonstrated that *Rph3* interacts with *Rph5* or an additional locus closely linked to *Rph5* (tentatively designated *RphGID*) in GID to produce an incompatible response when challenged with a pathotype virulent on *Rph3+Rph5*.

## Introduction

Leaf rust of barley, caused by *Puccinia hordei*, is the most common and prevalent disease in temperate barley-growing regions (Park et al., 2015). This disease can cause yield losses of up to 62% in susceptible cultivars under epidemic conditions (Clifford, 1985; Cotterill et al., 1992; Griffey et al., 1994; Park et al., 2015). Deploying host resistance has long been considered a cost-effective and environmentally friendly method to protect crop yield from this pathogen (Dinh et al., 2020). So far, 28 loci conferring resistance to leaf rust in barley have been characterized, viz. *Rph1* to *Rph28* (Mehnaz et al., 2021), of which 25 (*Rph1-Rph19*, *Rph21*, *Rph22, Rph25, Rph26, Rph27*, and *Rph28*) confer resistance at all growth stages (all stage resistance or ASR), and three (*Rph20*, *Rph23*, and *Rph24*) confer resistance at adult growth stages (adult plant resistance or APR) only. Notably, all the ASR genes conferring resistance to *P. hordei* identified so far are dominant. Up to date, only four out of 28 *Rph* genes have been isolated, with three genes encoding proteins of the Nucleotide-Binding Leucine-Rich Repeat (NLR) family (Dracatos et al., 2019; Wang et al., 2019; Chen et al., 2021) and one gene encoding protein of a distinct class (Dinh et al., 2022).

Several ASR *Rph* genes, including *Rph3*, *Rph7*, and *Rph9*, which were previously known to be effective have been extensively deployed in breeding programs globally (Park et al., 2015). However, the acquisition of virulence matching most of these major *Rph* genes by *P. hordei* has hampered their further utilization (Steffenson et al., 1993; Park et al., 2015; Kavanagh et al., 2017), and the deployment of such single major genes in large area can possibly result in serious epidemics (Brooks et al., 2000). Therefore, in parallel with mining new sources of resistance, deploying genes in combinations of two or more has proven to be an effective method of achieving a durable resistance against various pathogens (Kloppers and Pretorius, 1997; Fukuoka et al., 2015; Mundt, 2018). Interestingly, combinations of various “defeated” genes providing residual effects have been reported in multiple crops against different pathogens (Nass et al., 1981; Royer et al., 1984; Brodny et al., 1986; Li et al., 1999). The interaction of an APR and an ASR gene conferring resistance to *P. hordei* virulent pathotype has also been reported (Singh et al., 2021). When being singly deployed, both the ASR gene *Rph5.e* and the APR gene *Rph20* are ineffective against the pathotype 220 P+ +Rph13 at the seedling stages. However, the *Rph5.e* + *Rph20* combination stayed effective when challenged with the pathotype 220 P+ +Rph13. These findings suggested that the residual effect of “defeated” genes can confer the resistance to *P. hordei* virulent pathotype.

A barley breeding line GID 5779743 was among germplasm imported to Australia as part of the CAIGE (CIMMYT Australia ICARDA Germplasm Enhancement) project (CAIGE code 67:ZBS15; Australian Grains Genebank accession AGG411915BARL). Tests with the most prevalent pathotype of *P. hordei* in Australia, viz. 5457 P+, showed that it carried uncharacterized ASR to this pathogen. This study was undertaken to characterize the genetic basis of the resistance to *P. hordei* in GID 5779743.

## Materials and methods

### Plant materials and growing conditions

The barley line GID 5779743 (Pedigree: Shenmai No. 3/MSEL) investigated in this study is an accession from the CAIGE barley germplasm collection. The pedigree of Shenmai No. 3 is Shenmai No. 1/Humai No. 10, while the pedigree of MSEL is unknown. Other barley accessions carrying various *Rph* genes were used as controls (Park et al., 2015). GID 5779743 (here onward referred to as GID) was crossed to a susceptible line (Gus) to develop four F_2_ populations (POP1–POP4) derived from four F_1_ seeds. F_2_ plants of the population POP1 were advanced to the F_3_ generation for further studies.

Six *P. hordei* pathotypes designated according to the octal notation proposed by Gilmour (1973) (viz. 200 P− [Plant Breeding Institute culture number 518], 220 P+ +Rph13 [577], 5457 P+ [612], 5477 P− [672], 253 P− [490], and 5652 P+ [561]) were used in this study. The suffix P+/P− added to each octal designation indicated virulence/avirulence for resistance gene *Rph19* (Park, 2003). These pathotypes were originally raised from single uredinia on the leaf rust susceptible genotype cv. Gus in the greenhouse and the urediniospores were dried above silica gel for 5–7 days at 12°C before being stored in liquid nitrogen at the Plant Breeding Institute, The University of Sydney, Australia. Details for each pathotype, including pathogenicity on different resistance genes, are listed in the Supplementary Table 1.

### Phenotypic analysis

Seedlings were raised in 9-cm-diameter pots. The pots were watered with a soluble fertilizer (Aquasol^®^, Hortico Pty. Ltd., Revesby, NSW, Australia) at the rate of 35 g in 10 L of water for 100 pots, before sowing. Each F_3_ family was sown using 25–30 seeds/pot. Seedlings of differential lines and parents were raised by sowing clumps with 8–10 seeds of each.

Greenhouse inoculations were performed following the technique described by Golegaonkar et al. (2009). After sowing, the pots were transferred to rooms maintained at 23 ± 2°C with natural light, and seedlings were raised until ready for inoculations. The inoculations were carried out on 8–10 days-old seedlings with fully expanded first leaves using urediniospores (10 mg of spores per 10 ml of mineral oil per 200 pots) of *P. hordei*. The inoculated seedlings were placed in the incubation room for 24 h at ambient temperatures in a misted dark room where an ultrasonic humidifier generated the mist. The seedlings were moved to greenhouse chambers at 23 ± 2°C, and disease responses were recorded after 10 days, using a “0”–“4” infection type scale (Park et al., 2015). Infection types (ITs) of “2+” and lower were considered resistant, while “3” or higher indicated susceptibility.

### Genetic analysis

Four F_2_ populations (POP1–POP4) derived from four F_1_ seeds of the cross GID/Gus were subjected to phenotypic assays with four *P. hordei* pathotypes (POP1 with pt. 5457 P+, POP2 with pt. 220 P+ +Rph13, POP3 with pt. 200 P−, and POP4 with pt. 5477 P−) to examine the inheritance of the resistance against each.

A total of 415 F_3_ families from POP1 were inoculated with pathotypes 5457 P+ and 5477 P−, while 61 F_3_ families selected randomly from these 415 families were inoculated with 200 P− and 220 P+ +Rph13. The response to each *P. hordei* pathotype of these families was recorded 9 days after inoculation when the susceptible control line “Gus” showed the high IT response (“3+”). These F_3_ families were scored as either non-segregating resistant (NSR), segregating (SEG), or non-segregating susceptible (NSS). The obtained data were statistically analyzed by Chi-squared analyses (χ^2^) to confirm the goodness-of-fit of observed ratios to theoretical expectations.

### DNA isolation and marker analysis

The total DNA from leaf samples was extracted using the SDS method as previously described by Dinh et al. (2022). About 30 mg of the first leaf of each seedling was sampled into a 96-well collection tube (12 × 8 wells) containing two ball bearings and subjected to DNA extraction using an SDS method. To stabilize the DNA, 450 μl of extraction buffer including 0.1 M of Tris–HCl buffer (pH 8.0), 0.005 M EDTA buffer (pH 8.0), 0.5 M NaCl, 2-Mercaptoethanol (70 μl/100 ml buffer), and RNAse (100 μg/ml) were added to each sample before crushing. A TissueLyzer II (Qiagen, Hilden, Germany) at 25 Hz for 2 min was used for crushing the leaf material in the extraction buffer. The final mixture was then added with SDS solution (1.2% final concentration) to solubilize the proteins and lipids at 65°C for 60 min. The remaining proteins were precipitated by adding ammonium acetate 7.5 M to reach a final concentration of 2 M. The mixture was incubated at 4°C for 60 min, followed by centrifuging at 4,800 rpm (4,327 × *g*) for 10 min to separate debris and the aqueous phase. The upper phase containing genomic DNA was transferred to a new 96 well format plate and pelleted out by adding 100 μl of chilled isopropanol to 100 μl of supernatant. The pellet was washed twice using 100 μl of 70% ethanol before being slowly dissolved in 200 μl TE 0.1x buffer for 6 h for downstream applications.

Primer3Plus software^1^ was used to design PCR primers that were subsequently synthesized commercially (Sigma-Aldrich, NSW, Australia). Each 10 μl PCR contained 0.2 units of high-fidelity DNA polymerase (MyFi™, Bioline, NSW, Australia), 0.3 μM of each primer, 1x MyFi reaction buffer (Bioline, NSW, Australia), and 20 ng of genomic DNA. Thermocycling conditions consisted of an initial denaturation of 95°C for 10 min followed by 30 cycles of 94°C for 30 s, 55–60°C for 30 s, 72°C for 30 s, followed by a final extension at 72°C for 10 min. PCR products were digested [using a suitable endonuclease when required (Supplementary Table 2)] for 3 h under the recommended temperature. The digested products were monitored by electrophoresis on an agarose gel and visualized by staining with 6x GelRed^®^ (Biotium, California, CA, United States) (1.5 μl/100 ml agarose gel).

Specific markers to detect the presence/absence of *Rph3* (Dinh et al., 2022), *Rph7* (Dracatos, unpublished), and *Rph15* (Chen et al., 2021) were used (Supplementary Table 3).

### High throughput genotyping using DArTseq markers and quantitative trait loci analysis

Based on the phenotypic data recorded from F_3_ families inoculated with *P. hordei* pathotype 5457 P+, DNA samples extracted from F_2_ plants corresponding to NSR and NSS families were selected for medium density genotyping. The concentration of extracted DNA was measured using a NanoDrop 1000 spectrophotometer (Thermo Fisher Scientific, Waltham, MA, United States) and adjusted to approximately 80 ng/μl and sent to Diversity Arrays Technology (DArT) Pty. Ltd., Australia for whole-genome profiling. DArTseq silico and DArTseq SNP markers (>23K) were used for genotyping 80 samples (39 samples from each resistant and susceptible group and two parents). DArTseq SNP markers were scored “1” or “0” for the “presence” or “absence” of the marker, while heterozygous genotypes were scored “1/1.” SilicoDArTs are scored in a binary fashion, representing genetically dominant markers with “1” and “0” for the “presence” or “absence” of the restriction fragment with the marker sequence in the genomic representation of the sample. For both sets of markers, the “-” symbol represented missing data.

Genotypic data received from DArTs coupled with the response to pathotype 5457 P+ from 78 F_3_ families of POP1 were subjected to linkage analysis and mapping. Before analysis, both sets of DArT data consisting of DArTseq silico and DArTseq SNP were curated by removing markers with unknown positions, reproducibility of less than 95%, call rate less than 98%, or markers with more than 20% of missing values. After marker curation, 1,440 DArTseq silico markers and 1,440 DArTseq SNP markers were used separately for QTLs analysis with WinQTLcart software. The phenotypic data used for both analyses were converted from susceptibility and resistance into binary values “0” and “1,” respectively. For both analyses, the QTLs with LOD values higher than the threshold were considered and validated to identify resistance loci.

### Developing polymerase chain reaction-based markers for detailed mapping

Based on the physical position of the DArTseq markers, the sequences of annotated high confidence genes listed on the IPK database within the detected locus were used to develop PCR-based markers. Various pairs of primers were designed based on the sequence of Morex to amplify the genomic DNA fragment from both parents of the mapping population (GID 5779743 and Gus). These amplicons were then sequenced using Sanger sequencing (Australian Genome Research Facility Ltd., Victoria, Australia) and aligned to determine the SNP that could be used to nominate suitable restriction enzymes. All markers used were co-dominant and therefore could differentiate heterozygous and homozygous forms. Genomic DNA of all 415 F_2_ individuals, including 78 samples used for DArT genotyping, was used to construct the detailed map of the detected QTL. The genetic map was constructed using MapChart version 2.32 (Voorrips, 2002). Genetic distances were calculated using the Kosambi mapping function (Kosambi, 2008).

## Results

### Phenotyping and genotyping of GID

**FIGURE 1:**
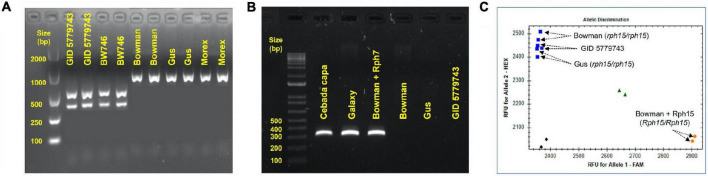
Genotype of GID 5779743 using various closely linked markers with known *Rph* genes. **(A)** The barley line BW746 is the NIL of Bowman carry *Rph3* that was used as the positive control. The *Rph3* gene is absent in the other barley cultivars, namely Bowman, Gus, and Morex, which were used as negative controls. **(B)** The absence of the resistance gene *Rph7* in the barley line GID 5779743 confirmed by marker genotyping. The dominant marker gives the amplicon of 319 bp in length when the gene is present in the barley cv. Cebada Capa, Galaxy, and Bowman+Rph7 while it is absent in three barley cv. Bowman, Gus, and GID 5779743. **(C)** The absence of the resistance gene *Rph15* in the barley line GID 5779743 was confirmed by marker genotyping. KASP marker illustrated the presence of *Rph15* in the barley NIL line Bowman+Rph15 by the orange dot in the bottom-right corner, the blue squares in the top-left corner demonstrated the absence of *Rph15* in three barley cv. Bowman, Gus, and GID 5779743, the green triangles showed the heterozygous form (*Rph15/rph15*) while the black diamonds in the bottom-left corner represented the water as no template control. All the PCRs were performed three time and generated similar results.

GID was resistant to all *P. hordei* test pathotypes while the IT responses of various leaf rust differentials varied from resistant to susceptible depending upon the pathotype used (Table 1). Based on multipathotype tests and IT response, the resistance in GID could be conferred by one of the known genes for which all pathotypes used were avirulent, such as *Rph7* or *Rph15*, combination of different resistance genes or a novel gene not reported yet. Genotyping GID with molecular markers linked to *Rph3, Rph7*, and *Rph15* suggested the presence of the *Rph3* (Figure 1A), but the absence of *Rph7* and *Rph15* (Figures 1B,C). Specifically, the genotypic data with the *Rph3* linked CAPS marker showed that GID had the same genotype as Bowman + *Rph3* NIL (Figure 1A), PCR with the *Rph7* linked marker did not generate any product (Figure 1B), and PCR with the *Rph15* linked KASP marker showed that GID had the same genotype as Gus and Bowman, which lack *Rph15* (Figure 1C). The phenotypic and genotypic analyses provided a strong evidence that GID carries the combination of *Rph3* and another resistance gene that was tentatively designated *RphGID*.

**TABLE 1:** Infection type* given by the parents (GID 5779743 and Gus) and controls with six *P. hordei* pathotypes.

Accession	Postulated	Pathotype [culture number]					
		
		5457 P+ [ = 612]	220 P + Rph13 [ = 577]	5477 P− [ = 677]	200 P− [ = 518]	253 P− [ = 490]	5652 P+ [ = 561]
GID	Unknown	0;n	0;n	;n	0;n	0;	0;n
Magnif	*Rph5*	0;n	gene 3+	3+	0;n	0;n	0;n
PI 531849	*Rph13*	;1n	3+	;1−n	0;	; −n	;n
Estate	*Rph3*	3+	0;	3+	;c	0;	0;
Cebada Capa	*Rph7*	;+cn	0;n	;c+	;n	;n	0;n
Gus	None	3+	3+	3+	3+	3+	3+

*Infection types recorded on a 0–4 scale (for details on scale refer Park et al., 2015).

### Genetic inheritance of the resistance in GID

Four F_2_ populations derived from four F_1_ seeds of the cross GID/Gus were challenged with various *P. hordei* pathotypes. The response of four F_2_ populations (POP1–POP4) revealed that two genes were involved in providing resistance to *P. hordei* in GID. The response observed in POP1 to pathotype 5457 P+ (virulent on *Rph3* and avirulent to *Rph5*) showed that a single locus (*RphGID*) conferred the resistance of GID to this pathotype (Tables 2, 3). Similarly, the response of POP2 to pathotype 220 P+ +Rph13 (avirulent on *Rph3* and virulent on *Rph5*) showed a single gene segregation, presumably due to *Rph3* (Table 2), and also demonstrated that *RphGID* is susceptible to this pathotype. The phenotypic data from POP3 challenged with *P. hordei* pathotype 200 P− (avirulent on *Rph3* and *Rph5*) showed segregation conforming to a digenic inheritance (Table 2) with likely involvement of *Rph3* and *RphGID*. The segregation in the response of POP4 to pathotype 5477 P− (virulent on *Rph3* and *Rph5*) suggested the involvement of two complementary genes (9 resistant: 7 susceptible; Table 2). The segregation ratio in the F_3_ generation of POP1 against the four pathotypes was consistent with those of the F_2_ populations (Table 3). The segregation of these four populations against various *P. hordei* pathotypes demonstrated the presence of two resistance genes, one being *Rph3* and the second gene considered *RphGID* at this stage.

**TABLE 2:** The inheritance of resistance to four pathotypes of *Puccinia hordei* in barley line GID 5779743 based on F_2_ populations derived from the cross GID/Gus.

POP	Pathotype	Number of F_2_ plants		Genetic ratio	χ^2^ (a)	*P*-value
		
		Resistant	Susceptible			
POP1	5457 P+	324	91	3:1	2.089	0.14835
POP2	220 P+ +Rph13	225	76	3:1	0.01	0.92048
POP3	200 P−	231	23	15:1	3.268	0.07068
POP4	5477 P−	94	76	9:7	0.063	0.80163

(a) χ^2^ table value at p = 0.05 is 3.84 (1 d.f.) and at p = 0.01 is 6.63 (1 d.f.).

**TABLE 3:** The inheritance of resistance to four pathotypes of *Puccinia hordei* in barley line GID 5779743 using F_2_-derived F_3_ families from the cross GID/Gus.

Pathotype	Response to rust			Genetic Ratio	χ^2^	*P*-value
	
	NSR	SEG	NSS			
5457 P+	103	221	91	1:2:1	2.45	0.29376
220 P+ +Rph13	15	31	15	1:2:1	0.02	0.99005
200 P−	24	33	4	7:8:1	0.85	0.65377
5477 P−	26	223	162	1:8:7	3.26	0.19593

χ^2^ table value at p = 0.05 is 5.99 (2 d.f.) and at p = 0.01 is 9.21 (2 d.f.). NSR, non-segregating resistant; NSS, non-segregating susceptible; SEG, segregating.

### Marker trait analysis

#### QTL conferring resistance to pathotype 220 P+ +Rph13

**FIGURE 2:**
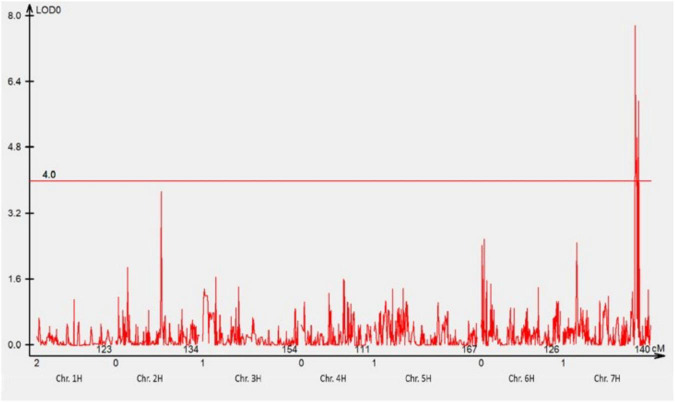
The most significant QTL conferring resistance to pathotype 220 P+ +Rph13 was detected using genotypic data of DArTseq Silico markers. One QTL was detected on the long arm of chromosome 7H. The threshold value was set at 4.0.

A total of 1,440 DArTseq Silico markers were retained after data curation. A total of 74 F_3_ families that were either NSR or NSS against pathotype 220 P+ +Rph13 were selected for QTL analysis. One QTL was detected at the *Rph3* locus on the distal region of chromosome 7HL (Figure 2) with the LOD = 7.8.

#### QTL conferring resistance to pathotype 5457 P+

**FIGURE 3:**
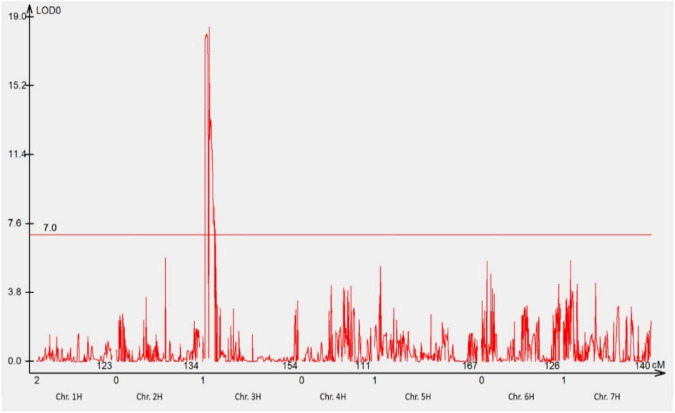
The most significant QTL conferring resistance to pathotype 5457 P+ detected using genotypic data of DArTseq SNP markers. One QTL was detected on the distal region of the short arm of chromosome 3H. The threshold value was set at 7.0.

The mapping population comprising of 78 F_3_ families was genotyped using 23,309 DArTseq silico and 11,458 DArTseq SNP markers covering the whole genome. After the data curation, 1,440 DArTseq SNP markers and 1,440 DArTseq silico markers were used together with the phenotypic data generated in phenotypic assay with pt. 5457 P+ for analyses. One locus on chromosome 3HS (*RphGID*) was detected with the LOD = 19, and 18 linked markers (Figure 3).

### Detailed map of *RphGID*

#### Pt. 5457 P+

**FIGURE 4:**
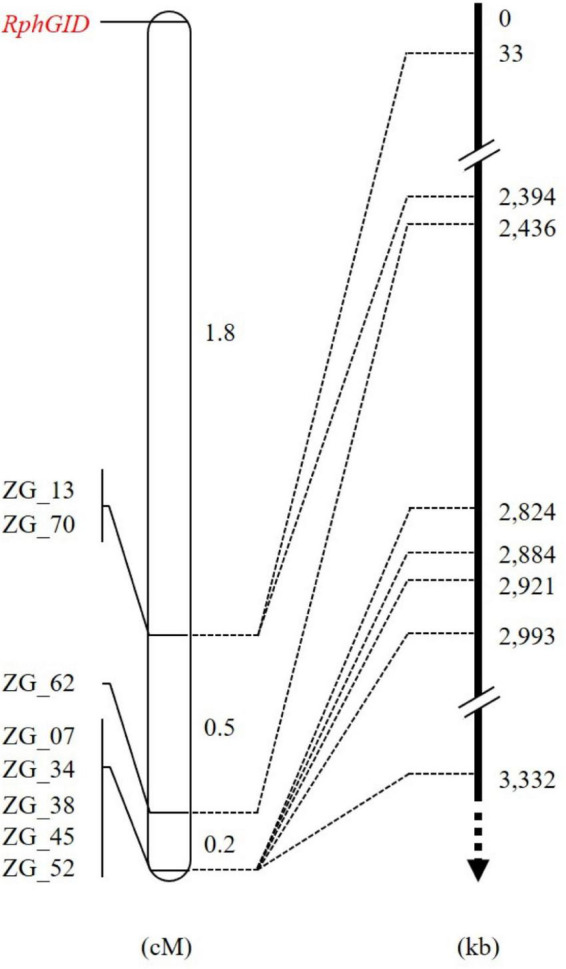
The detailed map of the *RphGID* locus. ZG_13 is the closest marker to the *RphGID* locus. The arrow is toward the centromere of chromosome 3H.

The whole F_2_ population consisting of 415 individuals was phenotyped with pt. 5457 P+ and genotyped using eight CAPS markers to construct a detailed map of the QTL *RphGID* (Supplementary Table 3). All of these markers resided on one side of the *RphGID* locus on chromosome 3H. The genetic distance from the closest marker ZG_13 to *RphGID* was 1.8 cM (Figure 4). Due to the telomeric location of the locus, the marker ZG_13 delimited locus *RphGID* in a physical window of 33 kb in length based on the reference genome of Morex v2.0 (2019). Notably, the *RphGID* was mapped to the same position as the *Rph5* locus, on the extreme telomeric region of chromosome 3HS.

#### Pt. 5477 P−

**FIGURE 5:**
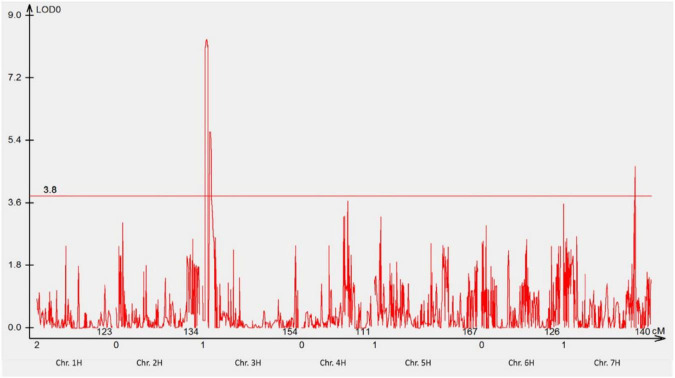
Manhattan plot showing the most significant QTL conferring resistance to pathotype 5477 P– using genotypic data of DArTseq silico markers. Two QTLs were detected, one QTL was located on the distal region of the short arm of chromosome 3H, and another one was located on the long arm of chromosome 7H. The threshold value was set at 3.8.

The response of POP4 and 411 F_3_ families derived from POP1 to *P. hordei* pathotype 5477 P− (Tables 2, 3) revealed the involvement of two genes interacting with each other in a complementary manner. Fifty-six lines showing either NSR or NSS responses to pt. 5477 P− were selected for QTL analysis. The analysis detected two major QTLs (on chromosomes 3HS and 7HL) involved in the resistance of GID to pt. 5477 P− (Figure 5). The position of linked DArT markers aligned with *RphGID* (3HS) and *Rph3* (7HL). Furthermore, the homozygous form of the *Rph3* gene was detected in all 26 F_2_ plants whose F_3_ families showed NSR responses to pt. 5477 P−. These results demonstrated the presence of the *Rph3* gene in GID and illustrated the involvement of this gene in the observed complementary interactions.

### Gene annotation for the *RphGID* locus

Most of the 33-kb delimited physical window sequence in the reference genome (cv. Morex) consisted of repetitive elements, and the FGENESH prediction tool showed five putative genes designated as *GID_ORF1* to *GID_ORF5* (Table 4). Among them, *GID_ORF1* encodes an unknown function protein without any conserved domains. Two conserved domains found in protein GID_ORF2, DEAD-like helicase and Helitron-like, were reported to be involved in ATP-dependent RNA or DNA unwinding. The MDN1 domain of GID_ORF3 was involved in ribosome maturation. The KOW motif of GID_ORF4 was known as an RNA-binding domain shared by some ribosomal proteins. The protein GID_ORF5 contains two conserved domains, namely RNase_HI_RT_Ty1 and RVT_2. The genomic DNA sequences of these five putative genes have been deposited in NCBI with the accession numbers OP021633, OP021634, OP021635, OP021636, and OP021637, respectively.

**TABLE 4:** Five putative genes were identified within the first 33 kb sequence of barley cv. Morex on the short arm of chromosome 3H.

Gene	Chain	DNA length (bp)	Protein length (aa)	Number of exons	Conserved motifs
*GID_ORF1*	−	2,661	253	5	None
*GID_ORF2*	+	6,616	1,184	12	DEAD-like_helicase_N, Helitron_like_N
*GID_ORF3*	+	1,362	351	4	MDN1
*GID_ORF4*	+	333	110	1	KOW
*GID_ORF5*	−	2,311	510	5	RNase_HI_RT_Ty1, RVT_2

## Discussion

This study combined multi-pathotype rust tests, marker genotyping, genetic analysis, and marker-trait associations to characterize leaf rust resistance in the ICARDA barley breeding line GID 5779743. The line was found to be seedling resistant to the most common pathotypes of *P. hordei* in Australia. Genotyping with closely linked markers demonstrated the presence of *Rph3* and the absence of *Rph7* and *Rph15* in this line. The genetic inheritance of resistance in four populations derived from a cross of GID/Gus revealed the involvement of two resistance genes. QTL analysis revealed that one of these two genes is *Rph3*, and the second (*RphGID*) is located in the extreme telomeric region of the short arm of chromosome 3H in the same genomic region as previously reported for *Rph5* (Mammadov et al., 2003). The low infection type produced by GID in response to pathotype 5457 P+ (avirulent on *Rph5*) was very similar to the typical low infection type (;+N) reported for gene *Rph5* (Park et al., 2015). The infection type patterns with multiple pathotypes, QTL analysis, and the high-resolution map of *RphGID* strongly suggested that *RphGID* is *Rph5*.

Interestingly, GID maintained its resistance when challenged with pathotype 5477 P− virulent on *Rph3* and *Rph5*. The genetic analysis of the F_2_ population and F_3_ families based on tests with this pathotype showed the involvement of two complementary genes mapped to the *Rph3* (7HL) and a locus on 3HS that can be *Rph5* or closely linked to *Rph5*. In addition, the detection of the *Rph3* gene in homozygous form in all 26 F_2_ plants whose F_3_ families showed no segregation responses to pt. 5477 P− demonstrated that the presence of *Rph3* in GID may be playing a role in conferring the resistance of GID to this pathotype. It is possible that *Rph3* interacts with another resistance gene, possibly *Rph5* (or another locus in the same genomic region) in a complementary fashion to produce an incompatible resistance response.

Combinations of some defeated genes can provide resistance to pathotypes with matching individual virulences, and these residual effects have been reported in multiple crops against different pathogens (Nass et al., 1981; Royer et al., 1984; Brodny et al., 1986; Li et al., 1999). In wheat, the interaction between *Yr73* and *Yr74* in a complementary fashion confers resistance to *Puccinia striiformis* f. sp. *tritici* (Dracatos et al., 2016), while complementary action between *Lr27* and *Lr31* provided resistance to *Puccinia triticina* in wheat (Singh et al., 1999). The complementary action conferring APR to *P. hordei* was also reported on the barley cv. Mecknes Maroc (Elmansour et al., 2017). Very recently, Singh et al. (2021) reported on the interaction of an APR (*Rph20*) and an ASR (*Rph5.e*) gene conferring resistance to *P. hordei* virulent pathotype. In soybean, the resistance of accession JS 95-60 to the fungus *Colletotrichum truncatum* was also regulated by the interactions of two major genes in complementary fashion (Nataraj et al., 2020). In the current study, we hypothesized that two defeated genes (*Rph3* and *RphGID/Rph5*) interact in a complementary fashion to produce resistance against a pathotype virulent on each gene individually. Allelic studies are recommended to further resolve the genetic basis of resistance in GID.

Most plant genes conferring resistance to pathogens encode proteins of the NLR family (Dodds and Rathjen, 2010; Zipfel, 2014). None of five putative genes identified by the FGENESH tool within 33-kb of the *RphGID* locus encodes a protein of the NLR family, and none were reported to be associated with the resistance to any plant diseases. Notably, the putative genes were identified based on the genome of barley cv. Morex, while Morex is susceptible to *P. hordei* pathotype 5457 P+, the *RphGID* locus may not exist in this accession. Many disease resistance genes isolated so far such as the genes conferring resistance to *Xanthomonas* bacteria in rice and pepper (Yoshimura et al., 1998; Wu et al., 2008; Wang et al., 2015, 2018), and especially the barley leaf rust resistance gene *Rph3* (Dinh et al., 2022) encode other types of protein without any conserved domains. In our study, we were not able to pinpoint whether all the identified genes in ORF region play role in providing resistance against all the six pathotypes used in this study and therefore further studies on validation of these genes is highly recommended.

In conclusion, the current study demonstrates that the barley line GID carries two independent loci, *Rph3* and *RphGID*. This study also illustrated that the interactions between *Rph3* and *RphGID* in a complementary manner confers the resistance to the *P. hordei* pathotype 5477 P−, which is virulent on *Rph3* when this gene is singly deployed. Based on the responses to various *P. hordei* pathotypes and the detailed genetic map, we suggest that *RphGID* can be *Rph5* or an allele of this gene. A test of allelism is required to validate this hypothesis. Besides, more efforts are needed to fine-map toward identifying the genetic sequences behind the resistance to *P. hordei* in the GID 5779743 line. The interactions between defeated genes to confer resistance to pathogens is also a prospect due to the limited number of identified resistance genes.

## Data availability statement

The datasets presented in this study can be found in online repositories. The names of the repository/repositories and accession number(s) can be found in the article/Supplementary material.

## Author contributions

DS and RP conceived the project, designed multi-pathotype tests, oversaw all rust phenotyping, and performed the gene postulation. DS provided the materials. RP provided all rust isolates and information on pathogenicities. HD performed multi-pathotype tests, genotyped the materials, and performed the QTL analysis. HD and MP designed the markers, analyzed the genotypic data, and constructed the high-resolution map. HD and DS wrote the manuscript. All authors reviewed the manuscript and approved the submitted version.

## Footnotes

1
http://www.bioinformatics.nl/cgi-bin/primer3plus/primer3plus.cgi

## References

[B1] Brodny U. Nelson R. Gregory L. (1986). Residual and interactive expressions of “Defeated” wheat stem rust resistance genes. *Phytopathology* 76 546–549. 10.1094/Phyto-76-546

[B2] Brooks W. S. Griffey C. Steffenson B. Vivar H. (2000). Genes governing resistance to Puccinia hordei in thirteen spring barley accessions. *Phytopathology* 90 1131–1136. 10.1094/PHYTO.2000.90.10.1131 18944477

[B3] Chen C. Jost M. Clark B. Martin M. Matny O. Steffenson B. J. (2021). BED domain-containing NLR from wild barley confers resistance to leaf rust. *Plant Biotechnol. J.* 19 1206–1215. 10.1111/pbi.13542 33415836 PMC8196641

[B4] Clifford B. (1985). “Barley leaf rust,” in *The cereal rusts Vol. II: Diseases, distribution, epidemiology and control’*, eds Roelfs A. P. Bushnell W. R. (Amsterdam: Elsevier), 173–205. 10.1016/B978-0-12-148402-6.50014-6

[B5] Cotterill P. Rees R. Platz G. Dill-Macky R. (1992). Effects of leaf rust on selected Australian barleys. *Aust. J. Exp. Agric.* 32 747–751. 10.1071/EA9920747

[B6] Dinh H. X. Singh D. Gomez de la Cruz D. Hensel G. Kumlehn J. Mascher M. (2022). The barley leaf rust resistance gene Rph3 encodes a predicted membrane protein and is induced upon infection by avirulent pathotypes of *Puccinia hordei*. *Nat. Commun.* 13 1–13. 10.1038/s41467-022-29840-1 35501307 PMC9061838

[B7] Dinh H. X. Singh D. Periyannan S. Park R. F. Pourkheirandish M. (2020). Molecular genetics of leaf rust resistance in wheat and barley. *Theor. Appl. Genet.* 133 2035–2050. 10.1007/s00122-020-03570-8 32128617

[B8] Dodds P. N. Rathjen J. P. (2010). Plant immunity: Towards an integrated view of plant–pathogen interactions. *Nat. Rev. Genet.* 11 539–548. 10.1038/nrg2812 20585331

[B9] Dracatos P. M. Barto< J. Elmansour H. Singh D. Karafiátová M. Zhang P. (2019). The coiled-coil NLR Rph1, confers leaf rust resistance in barley cultivar Sudan. *Plant physiol.* 179 1362–1372. 10.1104/pp.18.01052 30593453 PMC6446784

[B10] Dracatos P. M. Zhang P. Park R. F. McIntosh R. A. Wellings C. R. (2016). Complementary resistance genes in wheat selection ‘Avocet R’confer resistance to stripe rust. *Theor. Appl. Genet.* 129 65–76. 10.1007/s00122-015-2609-7 26433828

[B11] Elmansour H. Singh D. Dracatos P. M. Park R. F. (2017). Identification and characterization of seedling and adult plant resistance to Puccinia hordei in selected African barley germplasm. *Euphytica* 213:119. 10.1007/s10681-017-1902-8

[B12] Fukuoka S. Saka N. Mizukami Y. Koga H. Yamanouchi U. Yoshioka Y. (2015). Gene pyramiding enhances durable blast disease resistance in rice. *Sci. Rep.* 5 1–7. 10.1038/srep07773 25586962 PMC5379001

[B13] Gilmour J. (1973). Octal notation for designating physiologic races of plant pathogens. *Nature* 242 620. 10.1038/242620a0

[B14] Golegaonkar P. G. Singh D. Park R. F. (2009). Evaluation of seedling and adult plant resistance to *Puccinia hordei* in barley. *Euphytica* 166 183–197. 10.1007/s10681-008-9814-2

[B15] Griffey C. Das M. Baldwin R. Waldenmaier C. (1994). Yield losses in winter barley resulting from a new race of *Puccinia hordei* in North America. *Plant Dis. (USA)* 78 256–260. 10.1094/PD-78-0256

[B16] Kavanagh P. J. Singh D. Bansal U. K. Park R. F. (2017). Inheritance and characterization of the new and rare gene Rph25 conferring seedling resistance in Hordeum vulgare against Puccinia hordei. *Plant Breed.* 136 908–912. 10.1111/pbr.12535

[B17] Kloppers F. Pretorius Z. (1997). Effects of combinations amongst genes Lr13, Lr34 and Lr37 on components of resistance in wheat to leaf rust. *Plant Pathol.* 46 737–750. 10.1046/j.1365-3059.1997.d01-58.x

[B18] Kosambi D. D. (2008). “Kosambi’s function,” in *Encyclopedia of genetics, genomics, proteomics and informatics*, ed. Rédei G. P. (Dordrecht: Springer), 1066–1066.

[B19] Li Z.-K. Luo L. Mei H. Paterson A. Zhao X. Zhong D. (1999). A “defeated” rice resistance gene acts as a QTL against a virulent strain of *Xanthomonas oryzae* pv. oryzae. *Mol. Gen. Genet.* 261 58–63. 10.1007/s004380050941 10071210

[B20] Mammadov J. Zwonitzer J. Biyashev R. Griffey C. Jin Y. Steffenson B. (2003). Molecular mapping of leaf rust resistance gene Rph5 in barley. *Crop Sci.* 43 388–393. 10.1094/PHYTO.2003.93.5.604 18942983

[B21] Mehnaz M. Dracatos P. Pham A. March T. Maurer A. Pillen K. (2021). Discovery and fine mapping of Rph28: A new gene conferring resistance to *Puccinia hordei* from wild barley. *Theor. Appl. Genet.* 134 2167–2179. 10.1007/s00122-021-03814-1 33774682

[B22] Mundt C. C. (2018). Pyramiding for resistance durability: Theory and practice. *Phytopathology* 108 792–802. 10.1094/PHYTO-12-17-0426-RVW 29648947

[B23] Nass H. Pedersen W. MacKenzie D. Nelson R. (1981). The residual effects of some “Defeated” powdery mildew resistance genes. *Phytopathology* 71 1315–1318. 10.1094/PHYTO.1999.89.7.533 18944687

[B24] Nataraj V. Maranna S. Kumawat G. Gupta S. Rajput L. S. Kumar S. (2020). Genetic inheritance and identification of germplasm sources for anthracnose resistance in soybean [*Glycine max* (L.) Merr.]. *Genet. Resour. Crop Evol.* 67 1449–1456. 10.1007/s10722-020-00917-4

[B25] Park R. F. (2003). Pathogenic specialization and pathotype distribution of *Puccinia hordei* in Australia, 1992 to 2001. *Plant Dis.* 87 1311–1316. 10.1094/PDIS.2003.87.11.1311 30812545

[B26] Park R. F. Golegaonkar P. G. Derevnina L. Sandhu K. S. Karaoglu H. Elmansour H. M. (2015). Leaf rust of cultivated barley: Pathology and control. *Annu. Rev. Phytopathol.* 53 565–589. 10.1146/annurev-phyto-080614-120324 26047566

[B27] Royer M. Nelson R. MacKenzie D. Diehle D. (1984). Partial resistance of near-isogenic wheat lines compatible with *Erysiphe graminis* f. sp. tritici. *Phytopathology* 74 1001–1006. 10.1094/Phyto-74-1001

[B28] Singh D. Park R. F. McIntosh R. (1999). Genetic relationship between the adult plant resistance gene Lr12 and the complementary gene Lr31 for seedling resistance to leaf rust in common wheat. *Plant Pathol.* 48 567–573. 10.1046/j.1365-3059.1999.00391.x

[B29] Singh L. Park R. F. Dracatos P. Ziems L. Singh D. (2021). Understanding the expression and interaction of Rph genes conferring seedling and adult plant resistance to *Puccinia hordei* in barley. *Can. J. Plant Pathol.* 43(suppl. 2) S218–S226. 10.1080/07060661.2021.1936649

[B30] Steffenson B. Jin Y. Griffey C. (1993). Pathotypes of Puccinia hordei with virulence for the barley leaf rust resistance gene Rph7 in the United States. *Plant Dis.* 77 867–869. 10.1094/PDIS-08-12-0785-PDN 30722623

[B31] Voorrips R. (2002). MapChart: Software for the graphical presentation of linkage maps and QTLs. *J. Heredity* 93 77–78. 10.1093/jhered/93.1.77 12011185

[B32] Wang C. Zhang X. Fan Y. Gao Y. Zhu Q. Zheng C. (2015). XA23 is an executor R protein and confers broad-spectrum disease resistance in rice. *Mol. Plant* 8 290–302. 10.1016/j.molp.2014.10.010 25616388

[B33] Wang J. Zeng X. Tian D. Yang X. Wang L. Yin Z. (2018). The pepper Bs4C proteins are localized to the endoplasmic reticulum (ER) membrane and confer disease resistance to bacterial blight in transgenic rice. *Mol. Plant Pathol.* 19 2025–2035. 10.1111/mpp.12684 29603592 PMC6638055

[B34] Wang Y. Subedi S. de Vries H. Doornenbal P. Vels A. Hensel G. (2019). Orthologous receptor kinases quantitatively affect the host status of barley to leaf rust fungi. *Nat. Plants* 5 1129–1135. 10.1038/s41477-019-0545-2 31712760

[B35] Wu L. Goh M. L. Sreekala C. Yin Z. (2008). XA27 depends on an amino-terminal signal-anchor-like sequence to localize to the apoplast for resistance to *Xanthomonas oryzae* pv oryzae. *Plant physiol.* 148 1497–1509. 10.1104/pp.108.123356 18784285 PMC2577279

[B36] Yoshimura S. Yamanouchi U. Katayose Y. Toki S. Wang Z. Kono I. (1998). Expression of Xa1, a bacterial blight-resistance gene in rice, is induced by bacterial inoculation. *Proc. Natl. Acad. Sci. U.S.A.* 95 1663–1668. 10.1073/pnas.95.4.1663 9465073 PMC19140

[B37] Zipfel C. (2014). Plant pattern-recognition receptors. *Trends Immunol.* 35 345–351. 10.1016/j.it.2014.05.004 24946686

