# Supplementary material for: The genetic basis and interaction of genes conferring resistance to Puccinia hordei in an ICARDA barley breeding line GID 5779743
Source: Front Plant Sci. 2022 Aug 16;13:988322. doi: 10.3389/fpls.2022.988322 (PMC9425046; doi:10.3389/fpls.2022.988322)
Supplement: Supplementary file 1 [file Data_Sheet_1.PDF]

SUPPLEMENTARY TABLES

Table S1. Virulence profile and passport information of *Pucinia hordei* isolates used in this study

Pathotype	Species	Culture number	Year of collection	Place of collection	Virulence
200 P-	*Puccinia hordei*	518	not known	Queensland, Australia	*Rph8*
220 P+ +Rph13	*Puccinia hordei*	577	2003	South Australia	*Rph5*, *Rph8*, *Rph13*, *Rph19*
253 P-	*Puccinia hordei*	490	1976	South Australia	*Rph1*, *Rph2*, *Rph4*, *Rph6*, *Rph8*
5457 P+	*Puccinia hordei*	612	2009	Queensland, Australia	*Rph1*, *Rph2*, *Rph3*, *Rph4*, *Rph6*, *Rph9*, *Rph10*, *Rph12*, *Rph19*
5477 P-	*Puccinia hordei*	672	2017	South Australia	*Rph1*, *Rph2*, *Rph3*, *Rph4*, *Rph5*, *Rph6*, *Rph9*, *Rph10*, *Rph12*
5652 P+	*Puccinia hordei*	561	2001	Victoria, Australia	*Rph2*, *Rph4*, *Rph6*, *Rph8*, *Rph9*, *Rph10*, *Rph12*, *Rph19*

Table S2. DNA markers used to construct a detailed map of the *RphGID* locus

Marker name	Primer sequence	Chromosome	Physical position	Annealing temperature	Elongation time (s)	Expected size (bp)	Restriction enzyme
ZG_13	F: GGCTTGGAGCTCACTGAAAC<br>R: GTCAAGCTGTGCAATGGCTA	3HS	32213 - 33053	60	30	841	HpyCH4IV
ZG_70	F: GTTGAAACCCATGCGAAGAT<br>R: AGTGGCAGGTTGGAACAGAC	3HS	2397483 - 2396585	60	30	899	MseI
ZG_62	F: CACATTGGAACCAACACTCG<br>R: GTCCGTCCTTGGAAAATTCA	3HS	2436006 - 2435194	55	30	813	BstBI
ZG_07	F: ATTTTGGGGGTGAAGGAACT<br>R: GAACCCGAACTTGTGCATTT	3HS	2824173 - 2823291	60	30	883	HinfI
ZG_34	F: CAGAAAAGAGAGCCCAAACG<br>R: CATAGTCCCCACCGTTGAAG	3HS	2883976 – 2884875	60	30	900	AseI
ZG_38	F: GAAATTCCGCAAGGACAAAA<br>R: CGGGGTTTCTTCTTGTGTGT	3HS	2993551 – 2992709	60	30	843	BstBI
ZG_45	F: AAGCTTTGCAAACGGAAAGA<br>R: GGCATCAACGCTTAGGACAT	3HS	2921885 – 2921019	60	30	867	BsrI
ZG_52	F: GCTCCATGTATTTGCGGAGT<br>R: CAATGGCTCCTCCATCATCT	3HS	3331551 - 3330724	60	30	828	EcoRV

Table S3. Diagnostic markers used to genotype barley breeding line GID 5779743

Gene/Marker name	Primer sequence	Chr.	Physical position	Annealing temperature	Elongation time (s)	Expected size (bp)	Restriction enzyme
Rph3/MLOC_198	F: GCTGAGCCCCTAATACACGA<br>R: CCCATGTATGTGCTCGTTTG	7HL	612518564 - 612519434	55	30	870	BsrI
Rph15/KASP_15	A1: GAAGGTGACCAAGTTCATGCTGGGCTGTTATTAGCATGGTCCTC<br>A2: GAAGGTCGGAGTCAACGGATTGGGCTGTTATTAGCATGGTCCTG<br>C: AATACCACAATGACTACCCCAGGTT	2HS	43334316 - 43334415	57	30	99	N/A
Rph7	Dracatos et al (not published)						
